# Investigating SMR Peptide Interactions with Breast Cancer-Associated Proteins

**DOI:** 10.3390/ijms26188848

**Published:** 2025-09-11

**Authors:** Ming-Bo Huang, Purushottam B. Tiwari, Aykut Üren, Martin N. Shelton, Dara Brena, Jennifer Y. Wu, Mahfuz B. Khan, Michael D. Powell, Jonathan K. Stiles, Erica L. Johnson, Fengxia Yan, Lily Yang, Vincent C. Bond

**Affiliations:** 1 Department of Microbiology, Biochemistry, and Immunology, Morehouse School of Medicine, Atlanta, GA 30310, USAmkhan@msm.edu (M.B.K.); mpowell@msm.edu (M.D.P.); jstiles@msm.edu (J.K.S.); erijohnson@msm.edu (E.L.J.); vbond@msm.edu (V.C.B.); 2 Department of Oncology, Georgetown University Medical Center, Lombardi Comprehensive Cancer Center, Washington, DC 20057, USA; pbt7@georgetown.edu (P.B.T.); au26@georgetown.edu (A.Ü.); 3 Bruker Spatial Biology, Inc., 530 Fairview Ave. N., Seattle, WA 98109, USA; mshelton402@gmail.com; 4 School of International and Public Affairs, Columbia University, New York, NY 10027, USA; jw3573@columbia.edu; 5 Department of Community Health & Preventive Medicine, Morehouse School of Medicine, Atlanta, GA 30310, USA; fyan@msm.edu; 6 Department of Surgery and Radiology, Winship Cancer Institute, Emory University School of Medicine, Atlanta, GA 30322, USA; lyang02@emory.edu

**Keywords:** Vimentin, Mortalin, SMR peptide, peptide–protein interaction, surface plasmon resonance (SPR)

## Abstract

Breast cancer (BC) is a major cause of cancer-related mortality. Mortalin and Vimentin—two proteins implicated in BC progression and metastasis—have been identified as binding partners of the Secretion Modification Region (SMR) peptide from the HIV Nef protein. These interactions disrupt exosome release and offer novel therapeutic strategies. This study investigates the binding interactions between the SMR peptide, Mortalin, and Vimentin using surface plasmon resonance (SPR), co-immunoprecipitation (Co-IP), and Western blot assays. We also map the SMR binding sites on Mortalin through scanning peptide mapping and then identify a similar site on the Vimentin protein. Based on these data, we propose that the SMR peptide and its analogs interact with specific amino acid sequences in Mortalin and Vimentin, thereby disrupting cellular processes essential for Epithelial–Mesenchymal Transition (EMT) and tumor progression. SPR analysis revealed that the Nef protein exhibited the highest binding affinity to Vimentin (KD = 0.75 ± 1.1 nM) and Mortalin (KD = 3.16 ± 0.03 nM). The SMRwt peptide also demonstrated direct binding to both proteins with micromolar affinities (KD = 6.63 ± 0.74 µM for Vimentin; KD = 20.73 ± 2.33 µM for Mortalin), though the binding affinity was weaker than the full Nef protein. Co-IP experiments using MDA-MB-231, MCF-7, and BT474 BC cell lines confirmed that SMRwt, but not SMRmut, co-immunoprecipitated with Mortalin. Western blot analysis validated these interactions. Further, Mortalin peptide #56, derived from the substrate-binding domain, did not bind the SMR domain or inhibit Nef function. In contrast, peptides #61 and #62 from the C-terminal domain of Mortalin bound the SMR domain and effectively inhibited Nef activity. Notably, Mortalin peptide #61 inhibited SMRwt binding to both Mortalin and Vimentin, disrupting complex formation on the SPR sensor chip. These findings suggest that specific Mortalin-derived peptides can block SMR interactions, offering a potential therapeutic mechanism.

## 1. Introduction

Breast cancer (BC) is the second leading cause of cancer deaths in the United States. Triple-negative breast cancer (TNBC) is a clinically aggressive subtype of BC with high metastasis, tumor recurrence, and therapeutic resistance rates. Therefore, there is an urgent clinical need to understand the mechanism of action of anticancer drugs and avert BC metastases to improve survivability [1]. Exosomes are small extracellular vesicles (EVs) that are crucial in cell-to-cell communication. In the context of BC, tumor EVs (tEVs) have multiple significant functions, including tumor progression and metastasis [2,3,4,5], immune modulation [6,7], and drug resistance [3,4], and can have biomarkers for diagnosis and prognosis, with potential as therapeutic agents for the delivery of therapeutics [2,6]. In summary, BC tEVs play a pivotal role in the entire process [8,9,10,11,12,13,14].

Nef is an HIV protein (27 kDa) that enhances the Human Immunodeficiency Virus’s (HIV) ability to replicate and downregulate the expression of host cell surface receptors, such as CD4 and the chemokine receptor CCR5. Nef is produced early during HIV infection of a cell and is secreted from infected cells in exosomes (viral EVs, vEVs) [15]. Nef is a 27 kDa accessory protein encoded by HIV-1 that facilitates viral replication by downregulating host cell surface receptors such as CD4 and the chemokine receptor CCR5. Nef is produced early during HIV infection and is secreted from infected cells via exosomes [16]. The SMR motif, a five-amino-acid domain in Nef, is part of a complex that regulates the virus’s ability to manipulate the cellular trafficking pathway, releasing large numbers of vEVs containing Nef from infected cells [17]. Mutations in the SMR motif severely reduce or abolish HIV’s ability to release these Nef-containing vEVs [16]. The SMR motif is highly conserved across all HIV-1, HIV-2, and SIV clades, suggesting that this sequence is essential for the secretion process and important for the virus [18,19]. Given the Nef protein’s proclivity for interacting with cellular proteins to carry out its functions and this region’s sensitivity to even minor changes to its primary sequence, we hypothesized that the SMR constituted a binding site for some cellular factor(s) involved in Nef-induced vEV secretion and that disruption of this SMR-specific interaction would inhibit the virus’s ability to manipulate the cellular trafficking pathway, leading to a reduction in vEV secretion. In earlier published research, our lab identified Mortalin as one of the cellular proteins that interacts with the Nef SMR domain. Further, we showed that Mortalin is essential for vEV secretion, and that this interaction between Nef and Mortalin is susceptible to inhibition by a novel SMR-derived peptide. These findings strongly suggest that the SMR of Nef plays an important role in Nef’s ability to drive vEV secretion and helps explain this region’s sensitivity to changes.

In subsequent research, we further found that the SMR peptide can reduce the secretion of HER2-enriched tEVs in breast cancer (BC) cells. These tEVs are known to contribute to tumor aggressiveness and resistance to therapies such as Herceptin [19]. Additionally, we found that the SMR peptide blocks the release of tEVs, which have been implicated in tumor growth, immune evasion, and metastasis, in BC cells and in leukemia cells [20]. This SMR peptide-targeted reduction in tEV secretion suggests that the SMR peptide could be exploited as an anticancer agent [21,22].

We hypothesize that the SMR peptide modulates tEV formation and alters tEV cargo by directly interacting with target proteins, shifting the cargo away from the Epithelial–Mesenchymal Transition (EMT) program toward a more differentiated epithelial state (MET). This shift is expected to reduce the propensity for uncontrolled growth, invasion, and migration [21,23,24,25,26,27,28,29,30,31].

To this end, we used SPR to further examine the interactions between the SMR peptide and the two identified target proteins, Mortalin and Vimentin. We also performed dose–response experiments to determine the affinity and kinetic parameters of the interactions. The results of this study provide insight into how the SMR peptides modulate their target proteins, leading to disruption of EMT [32,33,34,35,36,37]. By understanding the interaction mechanism between SMR peptides and target sequences on key cancer proteins, new therapeutic strategies may be uncovered, targeting these proteins more effectively.

## 2. Results

### 2.1. SMRwt Peptide Interacts with Host Cell Proteins Mortalin and Vimentin in BC Cell Lines

**Figure 1:**
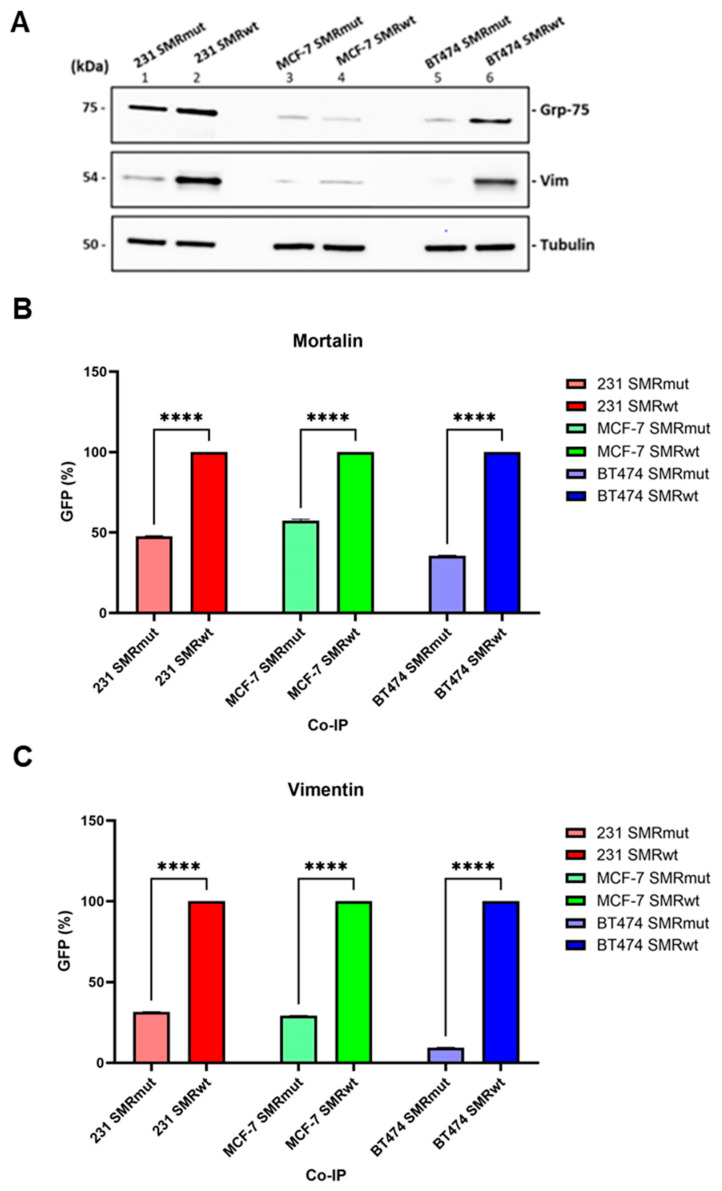
The SMRwt peptide specifically interacts with host cell proteins, including Mortalin and Vimentin. (**A**) Proteins from MDA-MB-231, MCF-7, and BT474 BC cells were co-immunoprecipitated with either SMRwt or SMRmut peptides using an anti-FLAG M2 antibody-coupled affinity resin. The procedure was also performed without the peptides as a control for nonspecific interactions. (**B**) Mortalin and (**C**) Vimentin. Densitometric analysis of Western blot data using the NIH ImageJ software 1.52 (NIH, Bethesda, MD, USA) (due to its sensitivity to pixel density and background correction) revealed significant differences: SMRmut vs. SMRwt **** *p* < 0.00005 in MDA-MB-231, **** *p* < 0.00005 in MCF-7, and **** *p* < 0.00005 in BT474 cells when probed with a Mortalin (Grp-75) antibody; **** *p* < 0.00005 in MDA-MB-231, **** *p* < 0.00005 in MCF-7, and **** *p* < 0.00005 in BT474 cells when probed with a Vimentin antibody.

In Figure 1, we investigate interactions between the SMRwt peptide and host cell proteins Mortalin (Grp-75) and Vimentin in BC cell lines. Understanding these interactions could provide insights into potential therapeutic targets for cancer treatment. The SMRwt peptide was bound to Mortalin and Vimentin in three BC cell lines: MDA-MB-231, MCF-7, and BT474. Co-immunoprecipitation assays were used to compare the binding efficiency of SMRwt and a mutant version, SMRmut, to these proteins. The results indicated that the SMRwt peptide explicitly interacts with Mortalin and Vimentin in all three tested BC cell lines. Of these, MDA-MB-231 and BT474 showed robust performance. Significant differences were observed between the SMRwt and SMRmut peptides, with SMRwt demonstrating higher levels of co-immunoprecipitation, suggesting more effective binding. Protein–Protein Interactions: The strong binding of SMRwt to Mortalin and Vimentin implies that these interactions are biologically relevant (Figure 1).

### 2.2. Peptide Sequence Motifs Specific to HIV Nef-SMR, Mortalin, and Vimentin: Roles in Protein Binding and Interaction

**Figure 2:**
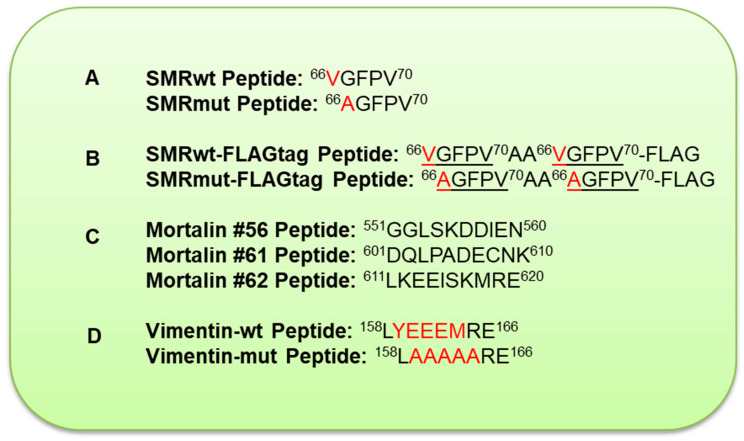
Differences between WT (wild-type) and MUT (mutant) peptides. (A) SMR peptides (HIV-1 Nef protein sequence has 206 AA): Schematic representation of the wild-type (SMRwt) and negative-control (SMRmut) synthetic peptides derived from HIV-1 Nef (206 AA) [19]. The difference: The first amino acid in the sequence changes from valine (V) in the WT peptide to alanine (A) in the MUT peptide. (B) SMR-FLAGtag peptides (HIV-1 Nef protein sequence has 206 AA): Schematic representation of the wild-type (SMRwt) and negative-control (SMRmut) synthetic peptides derived from HIV-1 Nef (206 AA) [19], showing sites of amino acid changes in SMRmut. Each peptide contains a FLAG tag at the C-terminus. The difference between SMRwt-FLAGtag and SMRmut-FLAGtag is that SMRwt-FLAGtag contains a valine (V) at the first position, while SMRmut-FLAGtag contains an alanine (A) at the first position. (C) Mortalin peptides: The Mortalin protein sequence has 679 AA. The sequences of Mortalin #61 and #62 show regions implicated in binding the HIV-1 Nef and SMRwt peptide. Mortalin #56 did not bind to Nef or the SMRwt peptide. The difference: These peptides are entirely different sequences, each representing different segments of the Mortalin protein. (D) Vimentin peptides: The Vimentin sequence has 466 AA. The difference between the Vimentin-wt peptide (LYEEEMRE) and the Vimentin-mut peptide (LYEEEMRE, red) lies in the substitution of the sequence YEEEM with AAAAA in the mutant peptide. This change significantly alters the peptide’s structure and function.

The sequences are labeled to show regions critical for binding interactions, particularly with SMR peptides. (A) HIV-1 Nef Protein and SMR Peptide: The HIV-1 Nef protein sequence (206 AA) has regions necessary for its interactions with other cellular proteins, and SMR motifs (specific regions within Nef) have been explored in earlier research [19]. (B) SMR Peptide Antagonists and SPR Analysis: Wild-type (SMRwt) and mutated (SMRmut) peptides with a FLAG tag were used to detect the binding affinity of different labeled SMR peptides. The point mutation in SMRmut altered the binding affinity, demonstrating the importance of specific amino acids in peptide–protein interactions. (C) Mortalin Protein and Mortalin Peptide: The Mortalin protein sequence has 679 AA, and two sequences (#61 and #62) show regions that bind HIV-1 Nef and the SMRwt peptide. Mortalin sequence #56 did not bind to Nef or the SMRwt peptide, indicating a lack of specific binding areas. Understanding these binding regions can be crucial for designing inhibitors or therapeutic agents targeting these interactions. (D) Vimentin Protein and Vimentin Peptide: The Vimentin sequence has 466 AA, and the sequence shows a specific area (158LYEEEMRE166) where SMR interaction occurs. This region might be involved in the structural organization of the cell and could be a target for intervention (Figure 2).

### 2.3. The Effects of Mortalin-Derived Peptides on the Secretion of EVs Containing Nef-GFP (exNef) on Three BC Cells and Jurkat Cells

**Figure 3:**
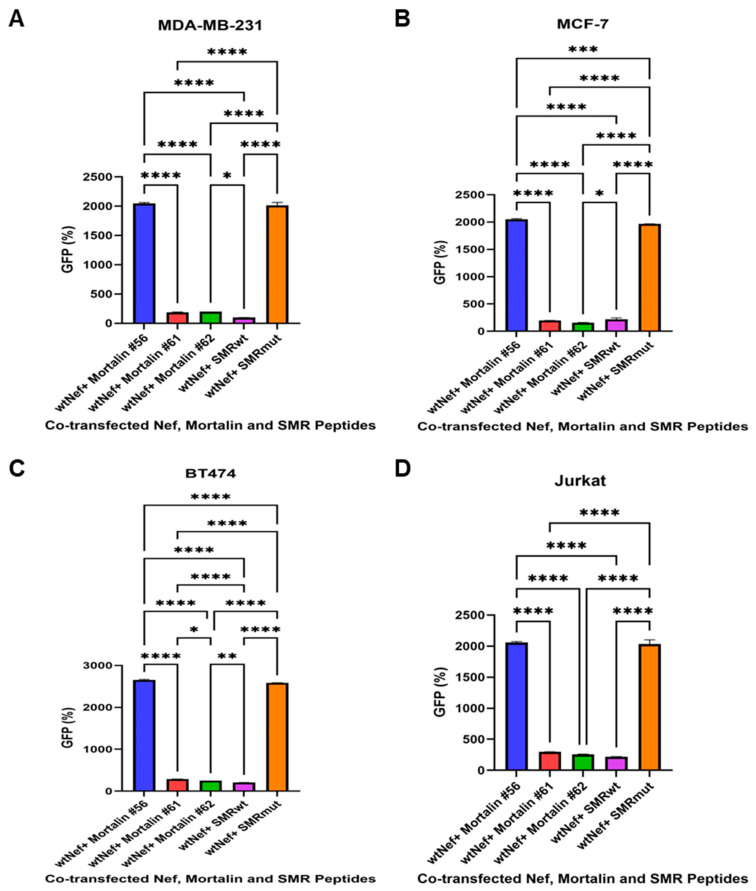
Effects of Mortalin-derived peptides on viral Nef-driven exNef secretion. (**A**) MDA-MB-231, (**B**) MCF-7, (**C**) BT474 BC cells, and (**D**) Jurkat T cells were co-transfected with 1 µg of wild-type Nef-GFP clone and 200 ng of various Mortalin-scanning peptides. The cells were then placed in a 24-well plate and incubated at 37 °C for 24 h before being assayed for extracellular vesicle secretion. Cells were also co-transfected with SMRwt or SMRmut peptides as positive and negative controls, respectively. The amount of GFP fluorescence measured was equivalent to the amount of exNef-GFP in the extracellular media. We determined that the Mortalin peptide #56 does not interact with the NefSMR domain and does not affect Nef function. In contrast, Mortalin peptides #61 and #62 can interact with the NefSMR domain and inhibit Nef function. The bar graphs display relative levels of exosome release. In here, asterisks (* *p* < 0.05, ** *p* < 0.005, *** *p* < 0.0005, and **** *p* < 0.00005) are used to indicate levels of statistical significance. The more asterisks, the stronger the statistical significance. Data represents the mean ± SD from three independent experiments.

The effects of Mortalin-derived peptides on the secretion of EVs containing Nef-GFP (exNef) were determined in three BC cell lines (MDA-MB-231, MCF-7, and BT474) and Jurkat T cells. Understanding these interactions could provide insights into potential therapeutic strategies against BC. The cells were co-transfected with Nef-GFP and various Mortalin peptides, including SMRwt and the control SMRmut peptides. The secretion of exNef-GFP was then measured to assess the impact of these Mortalin peptides on Nef function. The data shows that Mortalin peptide #56 did not block Nef function, indicating a lack of interaction with the *nef*SMR domain. Mortalin peptides #61 and #62 interacted with the *nef*SMR domain and successfully blocked Nef function, as evidenced by a significant reduction in exNef-GFP secretion. The ability of peptides #61 and #62 to block exNef secretion suggests that they specifically interact with the *nef*SMR domain, interfering with Nef’s function. This implies that the *nef*SMR domain is crucial for exNef secretion (Figure 3).

### 2.4. Impact of SMR, Mortalin, and Vimentin Peptides on Exosome Secretion in MDA-MB-231 and MCF-7 BC Cells

**Figure 4:**
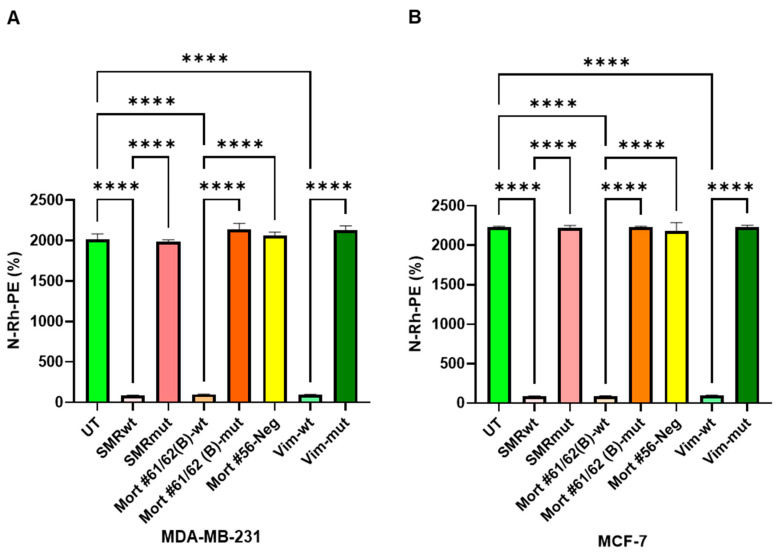
Effects of SMR, Mortalin, and Vimentin peptides on tumor cell exosome secretion in MDA-MB-231 and MCF-7 breast cancer cells. MDA-MB-231 and MCF-7 BC cells (200 µL, 1 × 10^4^ cells/mL) were seeded into a 96-well plate and treated with 100 ng/mL of either wild-type (wt) or mutant (mut) peptides of SMR, Mortalin, or Vimentin. Treatments were performed in RPMI-1640 medium without transfection, and cells were incubated at 37 °C for 24 h. The cells were then treated with 0.5 µM/mL of N-Rh-PE, incubated at 4 °C for 1 h, and washed with PBS. Fresh medium was also added. The cultures were incubated at 37 °C for an additional 24 h. N-Rh-PE refers to N-(Lissamine Rhodamine B sulfonyl) phosphatidyl-ethanolamine, a fluorescent lipid dye commonly used to label membranes and track vesicle trafficking. (**A**) MDA-MB-231 Cells: Data analysis of exosome release revealed significant differences: UT vs. SMRwt, Mort #61/62(B)-wt, VIM-wt **** *p* < 0.00005 in MDA-MB-231. (**B**) MCF-7 Cells: Data analysis of exosome release revealed significant differences: UT vs. SMRwt, Mort #61/62(B)-wt, VIM-wt **** *p* < 0.00005 in MCF-7. Exosome secretion was assessed by measuring the amount of N-Rh-PE, which corresponds to the number of exosomes released into the extracellular medium. Bar graphs show the relative exosome release level. Data represent the mean ± SD of three independent experiments.

Exosome secretion in MDA-MB-231 and MCF-7 BC cells was assessed by measuring the amount of N-Rh-PE, which corresponds to the number of exosomes released into the extracellular medium. Significant differences were observed in exosome release between untreated (UT) cells and those treated with wild-type peptides (SMR-wt, Mortalin-wt, and Vimentin-wt) in both MDA-MB-231 and MCF-7 cells. Treatment with wild-type peptides significantly decreased exosome release compared to untreated cells, indicating that these peptides may play a role in modulating exosome secretion. Figure 4 displays the relative levels of exosome release, with data representing the mean ± SD of three independent experiments. This visual representation clearly shows the differences in exosome secretion between the various treatments. Thus, Figure 4 highlights the potential impact of SMR, Mortalin, and Vimentin peptides on exosome secretion in BC cells, which could be crucial for developing new treatments targeting exosome-mediated pathways in cancer progression.

### 2.5. Mortalin Peptides Suppress exNef and Tumor Exosome Release

#### 2.5.1. Mortalin-Derived Peptides Inhibit exNef Secretion in Jurkat T Cells

**Figure 5:**
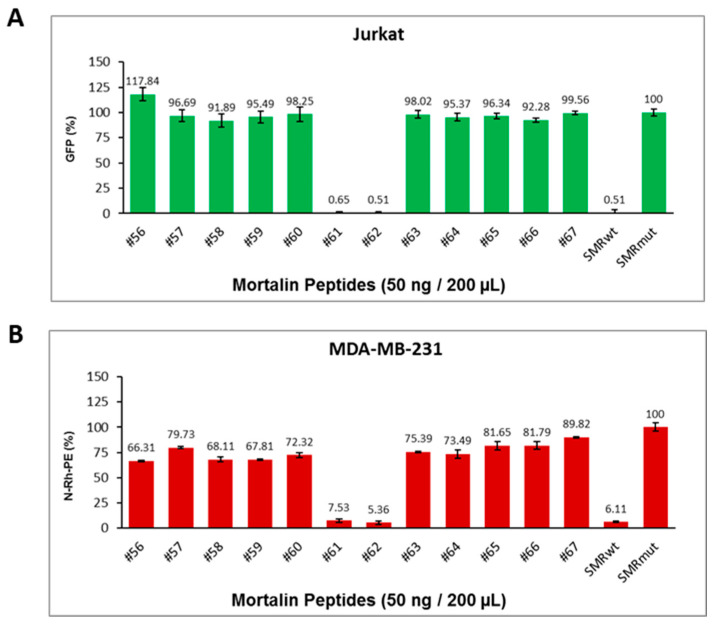
Effects of Mortalin-derived peptides on inhibition of exNef and tumor exosome secretion. (**A**) Jurkat T cells were co-transfected with 250 ng of wild-type Nef-GFP plasmid and 50 ng of Mortalin-derived scanning peptides (#56–#67), including SMRwt and SMRmut as positive and negative controls. After 48 h, GFP fluorescence in culture media was measured to assess exNef secretion. Data represent mean ± SD from three independent experiments. Statistical significance was determined using a two-sample *t*-test assuming equal variances. (**B**) MDA-MB-231 cells were transfected with 50 ng of Mortalin-derived scanning peptides (#56–#67), including SMRwt and SMRmut controls. Cells were labeled with N-Rh-PE and incubated for 48 h. N-Rh-PE fluorescence in culture media was measured to assess exosome secretion. Data represent mean ± SD from three independent experiments. Statistical significance was determined using a two-sample *t*-test assuming equal variances.

To determine whether Mortalin-derived peptides can inhibit the secretion of extracellular Nef (exNef), Jurkat T cells were co-transfected with wild-type Nef-GFP and various scanning peptides derived from the substrate-binding domain of Mortalin (#56–#67). SMRwt and SMRmut peptides were included as positive and negative controls, respectively. After 48 h of incubation, GFP fluorescence in the culture media was measured as a proxy for exNef secretion. As shown in Figure 5A, peptides #61 and #62 significantly reduced exNef secretion, with levels comparable to those observed with SMRwt. These peptides also formed small clusters, suggesting potential peptide aggregation or complex formation. Statistical analysis using a two-sample *t*-test assuming equal variances revealed highly significant differences: SMRmut vs. #61 (*p* < 1.47 × 10^−13^), SMRmut vs. #62 (*p* < 1.45 × 10^−13^), and SMRmut vs. SMRwt (*p* < 1.48 × 10^−13^).

#### 2.5.2. Mortalin-Derived Peptides Suppress Tumor Exosome Secretion in MDA-MB-231 Cells

To assess the effect of Mortalin-derived peptides on tumor exosome secretion, MDA-MB-231 breast cancer cells were transfected with the same panel of scanning peptides and labeled with N-Rh-PE to track exosome release. Following incubation, N-Rh-PE fluorescence in the culture media was measured as a proxy for extracellular exosome levels. SMRwt and SMRmut peptides were again used as controls. As shown in Figure 5B, peptides #61 and #62 significantly suppressed exosome secretion, mirroring the inhibitory effects observed in Jurkat cells. These peptides also formed small clusters. Statistical analysis confirmed significant differences: SMRmut vs. #61 (*p* < 7.20 × 10^−7^), SMRmut vs. #62 (*p* < 5.70 × 10^−7^), and SMRmut vs. SMRwt (*p* < 5.96 × 10^−7^).

### 2.6. Targeted Peptides Attenuate EV Release in Jurkat Cells and Breast Cancer Cells

#### 2.6.1. SMRwt and VIMwt Peptides Reduce Extracellular Vesicle Secretion in Jurkat T Cells

**Figure 6:**
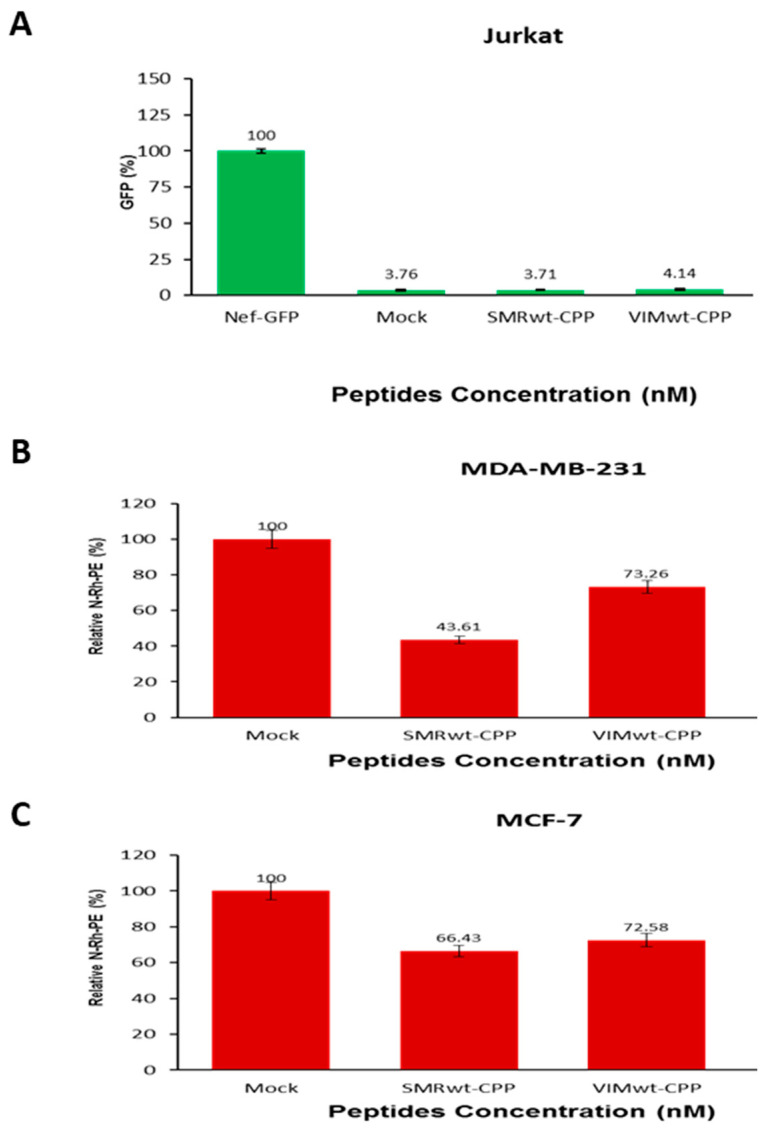
SMRwt and VIMwt peptides decrease extracellular vesicle secretion in Jurkat T cells and breast cancer cells. (**A**) Jurkat T cells were co-transfected with 1 µg of wild-type Nef-GFP plasmid and treated with either 349.6 nM SMRwt-CPP or 357.27 nM VIMwt-CPP peptides. After 24 h at 37 °C, culture media were collected and assayed for extracellular vesicle (EV) secretion using GFP fluorescence as a proxy. (**B**,**C**) MDA-MB-231 and MCF-7 breast cancer cells were treated with either 349.6 nM SMRwt-CPP or 357.27 nM VIMwt-CPP peptides for 24 h at 37 °C. Cells were then labeled with 5 µM N-Rh-PE at 4 °C for 1 h, washed, and incubated in fresh media for an additional 24 h. Supernatants were assayed for EV secretion using N-Rh-PE fluorescence. Bar graphs show relative EV levels. Data represent mean ± SD from three independent experiments. Statistical comparisons were performed using appropriate tests; *p*-values are indicated.

To investigate whether SMRwt and VIMwt peptides can suppress extracellular vesicle (EV) secretion, Jurkat T cells were co-transfected with wild-type Nef-GFP and treated with either SMRwt-CPP (349.6 nM) or VIMwt-CPP (357.27 nM) peptides. GFP fluorescence in the culture media was used as a proxy for Nef-associated EV release after 24 h of incubation. As shown in Figure 6A, both SMRwt-CPP and VIMwt-CPP treatments significantly reduced EV secretion compared to cells transfected with Nef-GFP alone. Statistical analysis revealed highly significant reductions in EV levels for both peptides (*** *p* < 0.0006), indicating their potential to inhibit Nef-mediated vesicle release.

#### 2.6.2. SMRwt and VIMwt Peptides Suppress EV Secretion in Breast Cancer Cells

To assess the effects of SMRwt and VIMwt peptides on EV secretion in breast cancer cells, MDA-MB-231 and MCF-7 cells were treated with either SMRwt-CPP (349.6 nM) or VIMwt-CPP (357.27 nM) for 24 h. Following treatment, cells were labeled with N-Rh-PE to track EV release, and supernatants were collected for fluorescence-based quantification. As shown in Figure 6B,C, both peptides significantly reduced EV secretion in MDA-MB-231 and MCF-7 cells compared to mock-treated controls. Statistical analysis confirmed that the reductions were significant: * *p* < 0.003 for MDA-MB-231 cells and * *p* < 0.0006 for MCF-7 cells. These findings suggest that SMRwt and VIMwt peptides broadly inhibit EV release across different cell types.

### 2.7. Comparative Analysis of Binding Affinity Between HIV-1 Nef, SMRwt/SMRmut Peptides, and Target Proteins Mortalin and Vimentin Using SPR

We conducted SPR experiments to characterize and quantify the direct binding interactions between Nef and SMR peptides (the analytes) and the target proteins, Vimentin and Mortalin (the immobilized ligands). We did so by injecting the analytes at various concentrations over the chip surface with the immobilized ligands.

**Figure 7:**
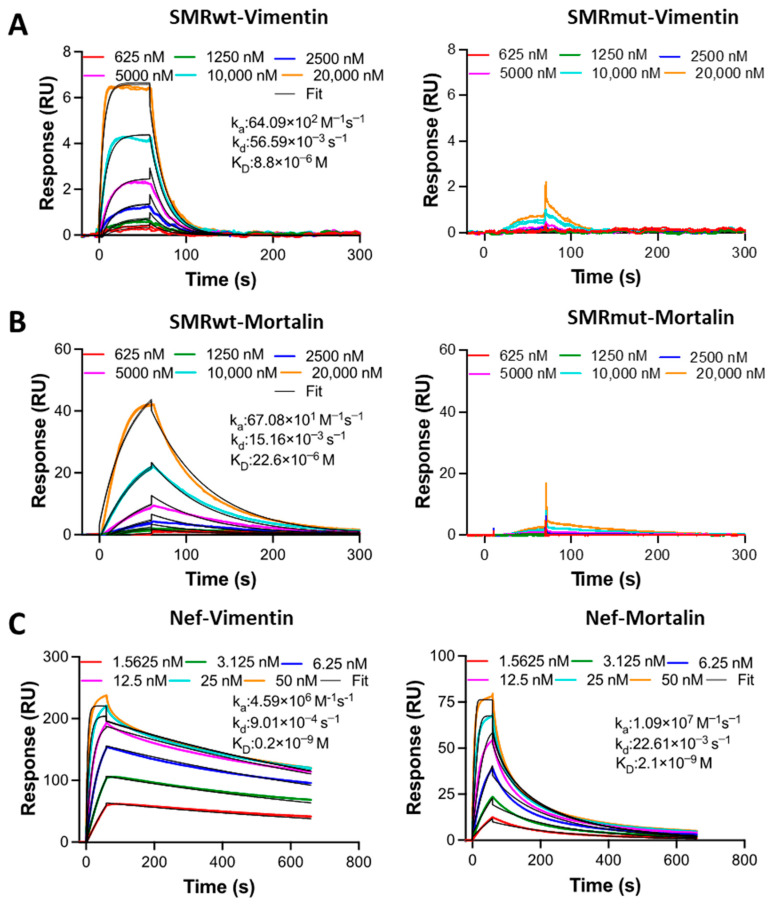
SPR analysis of SMRwt, SMRmut, and Nef binding to Vimentin and Mortalin. (**A**) Binding of SMRwt (**left**) and SMRmut (**right**) to immobilized Vimentin. (**B**) Binding of SMRwt (**left**) and SMRmut (**right**) to immobilized Mortalin. (**C**) Binding of Nef protein to immobilized Vimentin (**left**) and Mortalin (**right**). Colored lines represent experimental data, while black lines indicate fits to a 1:1 kinetics binding model. The derived kinetic parameters are displayed. Three independent experiments were performed, with one representative run shown here.

Table 1 and Figure 7 are the SPR results for Nef binding to Vimentin (left) and Mortalin (right). The colored lines are the experimental data. The Nef protein’s binding affinity to Vimentin was slightly stronger (lower K_D_ value) than that of Mortalin’s. The SPR results also show that the SMRwt peptide has a stronger affinity for Vimentin (Figure 7, left) than Mortalin (Figure 7, right). The panels on the right show that the SMRmut peptide did not produce a meaningful positive binding response to Vimentin and Mortalin, preventing the data from being fitted to derive K_D_ values. These SMRwt binding results (Figure 7, left) show that this peptide can compete with other Vimentin and Mortalin binding partners. Nef binds to both Vimentin and Mortalin.

Table 1 shows a comparison of analyte binding affinity (Nef protein and SMRwt/SMRmut peptides) to ligands (Vimentin and Mortalin). In SPR terminology, the immobilized biomolecule is referred to as the ligand, while the binding partner in the solution is called the analyte. This study utilized SPR to identify the complexes formed by SMR peptide proteins (Mortalin and Vimentin) and assess their binding affinity. The data are derived from three independent experiments.

### 2.8. SPR Kinetic Study of the Interaction Between Mortalin Peptides and Nef Reveals Differential Binding Affinity

**Figure 8:**
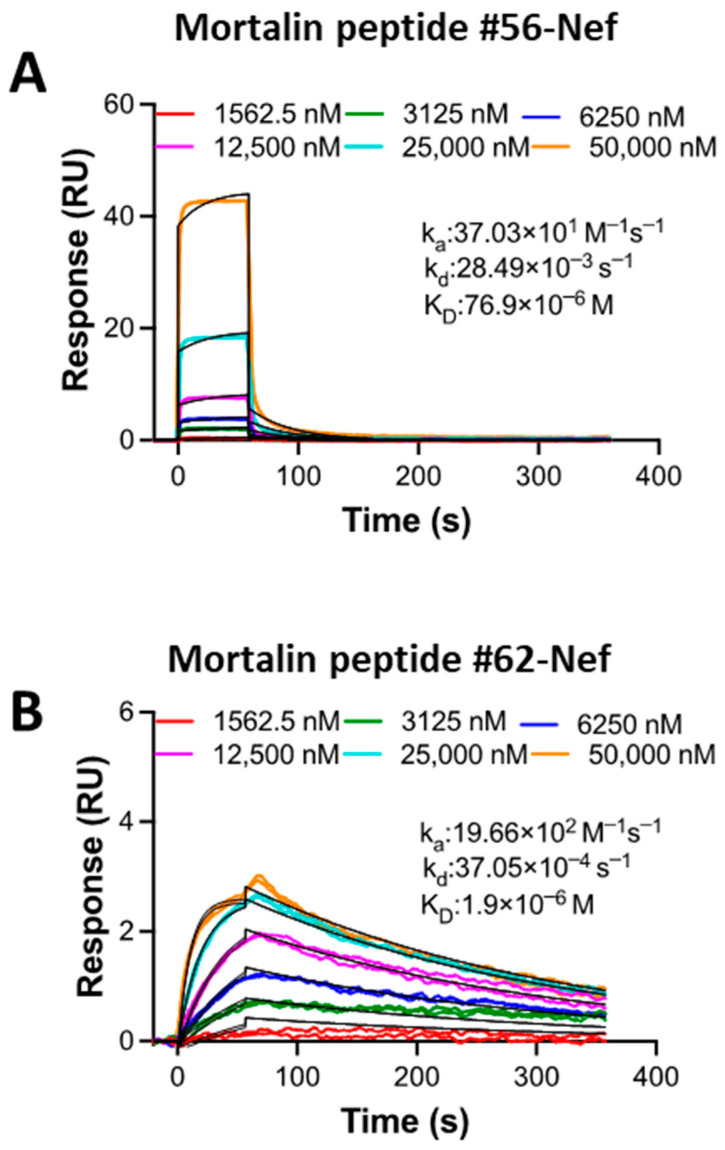
SPR results for the interaction between Mortalin peptides and the Nef protein. (**A**) Mortalin peptide #56 and (**B**) Mortalin peptide #62 binding to immobilized Nef protein, immobilized as a ligand on a CM5 sensor chip using thiol coupling chemistry. Colored lines represent experimental data, and black lines are fit to a 1:1 kinetics binding model. Derived kinetic parameters are shown. Mortalin peptide #62 had a much higher affinity for Nef binding than Mortalin peptide #56, evident from the 40-fold higher K_D_ of Mortalin peptide #56.

Figure 8 depicts the SPR results for Mortalin peptides binding to Nef. The Nef protein was immobilized as a ligand on the chip surface, and the peptides were used as analytes to flow into the buffer solution. Fitting of the SPR sensorgrams to a 1:1 kinetics binding model resulted in a weaker affinity (a higher K_D_ value) for Mortalin peptide #56 (Figure 8A) compared with the affinity value for Mortalin peptide #62 (Figure 8B) binding to immobilized Nef. The 40-fold difference in KD values between Mortalin peptides #56 and #62 for binding to Nef suggests that peptide #62 may be more effective at disrupting Nef’s interactions with other partners in associated signaling pathways.

### 2.9. SPR Analysis of Competition Binding Between SMR Peptide and Mortalin Peptides to Vimentin and Mortalin Proteins

**Figure 9:**
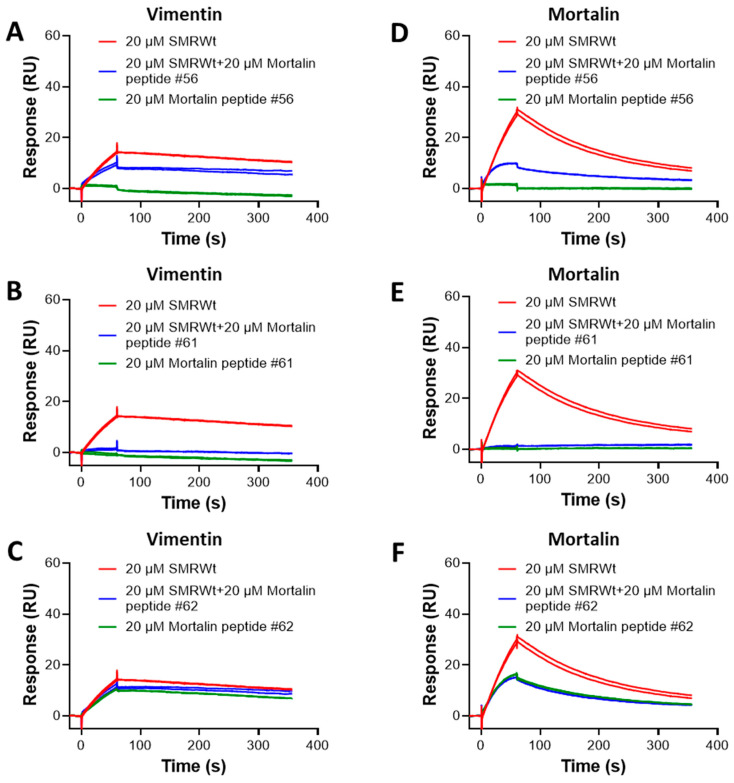
Competitive binding of SMR and Mortalin peptides to Vimentin and Mortalin proteins. Binding of SMRwt to immobilized Vimentin in the presence and absence of (**A**) Mortalin peptide #56, (**B**) Mortalin peptide #61, and (**C**) Mortalin peptide #62. Binding of SMRwt to immobilized Mortalin in the presence and absence of (**D**) Mortalin peptide #56, (**E**) Mortalin peptide #61, and (**F**) Mortalin peptide #62. SMRwt alone, as well as a mixture of His-tagged Mortalin peptides #56, #61, and #62 were injected as analytes to flow over the immobilized ligand surfaces. (**B**,**E**) show that Mortalin peptide #61 completely blocks the 20 µM SMRwt from binding to both Vimentin and Mortalin on the surface. By contrast, Mortalin peptide #56 (**A**,**D**) provides only partial blockage at 20 µM. In contrast to (**F**) for SMRwt binding to Mortalin, (**C**) shows no blockage of SMRwt binding to Vimentin. Vimentin and Mortalin proteins were immobilized as ligands on a CM5 sensor chip using standard amine coupling chemistry.

Figure 9 shows the SPR results for competitions between SMRmut and Mortalin peptides #56 and #62 for binding to Vimentin and Mortalin. Both Vimentin and Mortalin were immobilized as ligands. We used 20 µM of SMRwt alone and 20 µM of Mortalin peptides alone, and their mixtures were used as analytes.

While the competition experiments with Mortalin peptide #56 (Figure 9A) resulted in weak inhibition (~45%), 20 µM of Mortalin peptide #61 completely inhibited complex formation between SMRwt and Vimentin (Figure 9B). Furthermore, we also observed the nearly 70% inhibition of SMRwt–Mortalin complex formation in Mortalin peptide #56 (Figure 9D) and complete inhibition in the presence of Mortalin peptide #61 (Figure 9E). Since Mortalin peptide #62 at 20 µM shows binding to Vimentin (Figure 9C) and Mortalin (Figure 9F) comparable to SMRwt–Vimentin binding, we could not quantify the inhibition. However, if there were no competition, the signal amplitude for either SMRwt or Mortalin peptide #62 could be the same. The complete competition observed with Mortalin peptide #61 suggests that this peptide is a better candidate, compared with Mortalin peptides #56 and #62, for disrupting SMR-WT-related pathways, potentially in conditions where SMR-WT interaction with Mortalin or Vimentin plays a role.

### 2.10. Competitive Binding of Mortalin Peptides with SMRwt: Implications for Mortalin and Vimentin Interactions

**Figure 10:**
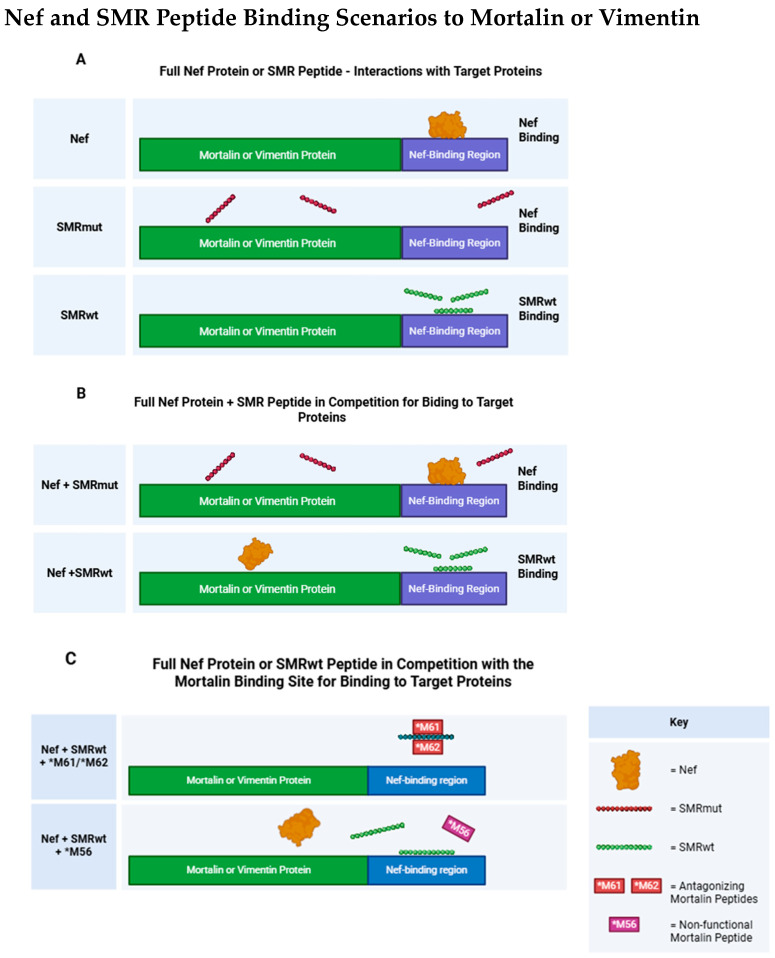
Binding scenarios involving Mortalin peptides competing with the SMR peptide, and the resulting effects on interactions between SMRwt and either Vimentin or Mortalin. (**A**) Full Nef protein or SMR peptide interactions with target proteins. (**B**) Full Nef protein + SMR peptide in competition for binding to target protein. (**C**) Full Nef protein or SMRwt peptide in competition with the Mortalin binding site for binding to target protein. Created with BioRender Brena, D (2025). https://BioRender.com/042t0rb (accessed on 5 September 2025).

Figure 10 shows scenarios where Nef and SMR peptides interact with Mortalin or Vimentin.

Mortalin #61 and #62 can interact with and effectively compete with SMRwt to block its interaction with the Mortalin or Vimentin protein. This suggests that Mortalin #61 and #62 contain specific sequences or motifs that recognize and bind to the SMRwt peptide, thereby preventing it from binding to its usual targets (Mortalin or Vimentin). However, the Mortalin #56 peptide does not interact with SMRwt and, therefore, cannot block SMRwt’s interaction with the Mortalin or Vimentin protein. This indicates that the #56 peptide lacks the sequence or structure required for binding to SMRwt, highlighting the specificity of its interaction with certain regions of Mortalin. The interaction between SMRwt and the Mortalin protein indicates that the native or unmutated sequence of SMRwt is recognized by Mortalin, enabling the formation of a protein–peptide complex. SMRmut has a point mutation (valine to alanine at the first amino acid from the N-terminus) and does not bind to the Mortalin protein, suggesting that the amino acid at the N-terminus is critical for the interaction between SMRwt and Mortalin. The mutation likely disrupts the peptide’s structure or its recognizability by Mortalin. These results underscore the importance of specific amino acid sequences and structural motifs in protein–peptide interactions. The ability of Mortalin #61 and #62 to compete with SMRwt indicates that these regions are crucial for binding. Conversely, the inability of Mortalin #56 and SMRmut to interact highlights the specificity of these interactions (Figure 10).

### 2.11. Development and Testing of Mortalin-Derived Scanning Peptides for Effects on EV Secretion

To identify peptide sequences within Mortalin that modulate extracellular vesicle (EV) secretion, a panel of scanning peptides was synthesized and tested in Jurkat T cells. The peptides, listed in Table 1, were custom-synthesized with an average purity of 75% and used in transfection assays to evaluate their impact on EV release. Jurkat T cells (3 × 10^5^) were co-transfected with 50 ng of individual Mortalin peptides or 250 ng of wild-type Nef-GFP plasmid using Chariot™ transfection reagent. Transfection efficiency was confirmed by fluorescence microscopy after 48 h. EV secretion was assessed by measuring GFP fluorescence in the culture media, and statistical analysis was performed using unpaired two-factor *t*-tests (*p* < 0.05). The peptide sequences and experimental conditions are summarized in Table S1. Panel of Mortalin scanning peptides developed and tested for their effect EV secretion and Table S2. Mortalin Scanning Peptides Used in Transfection Assays, see the Supplementary Information. All peptides were synthesized with an average purity of 75% and used at 50 ng per transfection in Jurkat T cells. Transfection efficiency was assessed by fluorescence microscopy, and EV secretion was quantified using GFP fluorescence. Statistical analysis was performed using unpaired two-factor *t*-tests (*p* < 0.05). 

### 2.12. Identification of a Vimentin-Derived Peptide with Homology to the Mortalin SMR Binding Sequence

To identify a Vimentin-derived peptide with potential functional similarity to the Mortalin SMR binding sequence, computational search was performed using the Mortalin SMR motif (LKEEISKMRE) as a query. The search revealed a homologous sequence within the human Vimentin protein, highlighted in Table S3, which closely resembles the Mortalin template. See the Supplementary Information. Based on this alignment, the corresponding Vimentin peptide was custom-synthesized by InnoPep (San Diego, CA, USA) for use in subsequent transfection and EV secretion assays. See the Supplementary Information.

## 3. Discussion

This study and the Supplementary Materials reveal several significant findings regarding the interactions between Nef, the SMR peptide, and the target proteins [38,39,40,41,42,43,44,45,46]. First, in the SPR analysis, Nef exhibits high affinity for Mortalin (2.1 nM) and Vimentin (0.2 nM), suggesting a crucial role in modulating these proteins’ functions within the cellular environment. Alternatively, the SMRwt peptide, which is one binding domain in the N-terminus Nef sequence, in past work has been shown to be part of an larger region involved in Nef-driven effects on the cellular trafficking pathway [19]. Structural modeling of the Nef SMR domain has previously been described and published [17]. This foundational work supports the rationale for targeting the SMR domain with peptide-based inhibitors and provides structural context for interpreting the effects of Mortalin- and Vimentin-derived peptides on exNef and EV secretion. The SMRwt peptide alone displays a lower affinity for Mortalin than the Nef protein. It also displays a higher affinity for Vimentin (6.63 ± 0.74 µM) versus Mortalin (20.73 ± 2.33 µM), to which it has a lower affinity. Thus, determining the affinity and kinetics of these interactions through SPR analysis has been fundamental to understanding the Nef protein versus the SMR peptide binding behavior at the cellular level. And, for the SMR peptide, this understanding is crucial for elucidating cancer metastasis mechanisms and identifying additional anticancer targets [47,48].

Providing insights into the structure–function relationships of the SMR peptide and its ability to bind specific target proteins, structural and functional studies of Mortalin have already identified the SBD of Mortalin, approximately spanning residues 400–600. This region includes a β-sheet sandwich core (~400–540) and a helical “lid” (540–600) that regulates substrate access; [49,50].

The C-terminal region (600–679) of Mortalin is not part of the core substrate-binding domain (SBD), but it plays regulatory and localization roles. These include co-chaperone interactions with co-chaperones like GrpE or Hsp40, modulating ATPase activity, mitochondrial targeting contributing to mitochondrial retention or localization, and protein–protein interactions suggested by previous observations that some antibodies (e.g., A305-256A-M) target this region, suggesting that it is surface-exposed and functionally relevant [50]. This C-terminal region is strongly hydrophilic, with repeating lysine (K) and glutamic acid (E) residues and no significant hydrophobic patches, suggesting it is not optimized for direct binding to hydrophobic regions of unfolded proteins. But this domain may still be structurally flexible, allowing transient exposure of hydrophobic patches or charged pockets that accommodate the binding of Nef or the SMR peptide, and could act as a regulatory interface, where SMR peptide binding disrupts Mortalin’s conformation, preventing co-chaperone docking or substrate handoff. It could also serve as a signal relay zone, where binding of the SMR domain alters Mortalin’s interactions with trafficking machinery. However, the 600–679 region of Mortalin is hydrophilic, flexible, and surface-exposed—ideal for acting as a recruitment platform extending outward from the protein’s core, functioning like a molecular “hand” to scan for specific unfolded proteins, recognizing exposed hydrophobic motifs, and guide them into the SBD for proper folding.

The Vimentin SMR binding sequence falls within the Rod Domain. Since proteins are synthesized from N-terminus to C-terminus, this region is translated after the Head Domain and early within the Rod Domain, making it accessible to chaperones like HSC70 during or shortly after synthesis. The Mortalin and Vimentin sequences are negatively charged and hydrophilic, suggesting surface exposure and potential for protein–protein interactions. The secondary structure of the sequences favors alpha-helical and turn structures, typical of flexible binding motifs. VIM shows a high alignment score to Mortalin’s domain (0.70), indicating sequence-level mimicry. And these motifs seem to represent conserved acidic patches used for chaperone recognition, exosome trafficking, or EMT regulation.

The SMR is a compact, hydrophobic motif, likely to interact with hydrophilic or charged regions via hydrophobic insertion into transiently exposed pockets, π-stacking or van der Waals interactions (especially via F), and conformational disruption due to the Proline residue. The SMR domain, being compact and hydrophobic, could mimic the hy-drophobic patches of unfolded proteins. Thus, the SMR peptide likely mimics a natural substrate motif or co-chaperone interface, flooding the mortalin C-terminal domain “hand”, blocking substrate recognition, modulating substrate delivery to the SBD, and disrupting co-chaperone docking, mediated through this same region. Further, the ability of these domains to compete for binding against each other suggests that there is some sequence specificity in addition to interactions due to general charge and hydrophobicity. Our data suggests that these sequences are critical functional interfaces, possibly involved in substrate guidance, co-chaperone interaction, and trafficking regulation, which aligns with our “extended hand” model. And the SMR peptide could be a multi-target trafficking disruptor, hijacking (in the case of the Nef protein) or blocking key protein–protein interfaces, disrupting the coordination between chaperones and their target proteins, and preventing proper cargo loading or vesicle movement.

On screening the Eucaryotic Linear Motif (ELM) database, which specializes in identifying short linear motifs (SLiMs) [51,52] that are functionally annotated, experimentally validated, and often involved in protein–protein interactions, trafficking, post-translational modifications, and signal transduction, it appears that the Mortalin and Vimentin sequences, while not exact matches to a known ELM, share features with several acidic, negatively charged motifs found in ligand-binding motifs (e.g., SH3, PDZ, FERM domains); trafficking motifs (e.g., NES, endosomal sorting signals); chaperone interaction motifs (e.g., HSP-binding regions); and FERM domain-binding motifs. Notable publications on ELM include ELM—Eukaryotic Linear Motif Resource, published in Nucleic Acids Research [53], which provides a comprehensive update on the ELM database, highlighting new features and expanded motif coverage [54]; the ELM 2016—Data Update and New Functionality, Nucleic Acids Research [54], which focuses on improvements in motif annotation and integration with other bioinformatics tools; and the ELM—the Eukaryotic Linear Motif Resource—2024 Update, Nucleic Acids Research [55], the latest update, highlighting new motif classes and enhanced search capabilities. Motifs with similar charge and structure are frequently found in proteins involved in EMT (Snail, Vimentin, Myosin-10), exosome biogenesis/release (ALIX, TSG101), and viral trafficking (HIV Nef, SARS-CoV-2 Spike) and are often located in disordered regions, making them accessible to chaperones.

Human viruses, in general, and in this case, HIV in particular, have evolved to operate efficiently on infection of higher organism cells (monkeys, and humans). They have picked up and used cellular genes, or cellular gene sequences, and incorporated them into use for their own purposes to manipulate cellular pathways.

HIV manipulates the cellular trafficking pathway as a way of making and releasing (i) viral particles, and (ii) in modulating and releasing inflammatory exosomes that disrupt the immune system and deter that system from attacking the virus-infected cells [56,57,58,59]. Thus, HIV viral particles have cloaked themselves as virus-modified exosomes using the cellular trafficking pathway for the above purposes Trojan Exosome Hypothesis [60].

There is a body of evidence that HIV Nef has an important role in manipulating the cellular trafficking pathway, and our lab has contributed to that literature. We have dissected the mechanisms of how, through a set of domains in the N-terminus of the Nef protein, the virus can manipulate the cellular trafficking pathway to release both viral particles and inflammatory virus-induced exosomes. However, there seems to be no evidence that there is a cellular homolog to Nef that functions similarly. Alternatively, we suggest that the virus has incorporated sequence domains into Nef (e.g., the secretion modification region/SMR) that can interact with and manipulate target cell proteins to do those functions described above. And we have published evidence that those target cellular proteins manipulated by Nef are proteins that are similarly manipulated by the tumorigenesis process, leading to EMT and cancer.

The Nef VGFPV domain is a viral domain highly conserved across all HIV clades. with a similar domain in SIV Nef also highly conserved across SIV clades. The data suggests that this Secretion Modification Region (SMR) may be an example of viral sequence mimicry targeting acidic, disordered motifs across multiple cellular proteins involved in exosome trafficking, EMT signaling, and chaperone-mediated folding [47,49].

### Conclusions and Future Directions

In this study, we identified and characterized novel peptide inhibitors that modulate extracellular vesicle (EV) and exNef secretion, with implications for both cancer and HIV pathogenesis. Our key findings include (i) the discovery of Mortalin-derived peptides (#61 and #62) that significantly suppress exNef and EV release in Jurkat T cells and breast cancer cell lines; (ii) demonstration of broad-spectrum EV inhibition by SMRwt and VIMwt peptides across multiple cell types, highlighting their potential as versatile modulators of vesicle trafficking; and (iii) identification of a Vimentin-derived peptide with sequence homology to the Mortalin SMR domain, reinforcing the functional relevance of this conserved motif in mediating protein–protein interactions. This work has provided key evidence supporting our initial published hypothesis (PMID23852082) that these virus domains can be used to identify functional domains on key cellular target proteins. And those identified cell protein domains can then be used to map functional interactions with other cellular proteins. And these small peptides can be used as targeted modulators of cellular function. Our data also support a model in which Nef, Mortalin, and Vimentin form a functional axis that regulates vesicle biogenesis and release. Thus, these findings underscore the therapeutic potential of targeting SMR-mediated interactions to disrupt pathogenic EV signaling. Our data also supports a model in which Nef, Mortalin, and Vimentin form a functional axis that regulates vesicle biogenesis and release.

Looking ahead, several avenues will be pursued to deepen our understanding and advance translational applications: (a) in vivo validation of peptide efficacy using relevant models of breast cancer and HIV infection; (b) mechanistic dissection of SMR-mediated signaling pathways, particularly those linked to cytoskeletal dynamics, stress responses, and vesicle trafficking, with efforts leveraging proteomic and phospho-proteomic profiling, pathway-specific reporter assays, and CRISPR-based perturbation studies; (c) optimization of peptide delivery strategies, including nanoparticle encapsulation and cell-penetrating modifications, to enhance bioavailability and tissue targeting.

Together, these future directions aim to clarify the biological significance of SMR interactions and lay the groundwork for novel therapeutic strategies targeting EV-mediated communication in cancer and infectious disease.

## 4. Materials and Methods

### 4.1. Cell Culture

MDA-MB-231, MCF-7, and BT474 are human BC cell lines. They are commonly used for cancer research and have been widely studied. MCF-10A is an epithelial cell line that was isolated in 1984 from the mammary gland of a 36-year-old white female with fibrocystic breasts. It is used as a model for normal breast tissue and often as a control for comparison with cancer cell lines. All these cell lines were purchased from the American Type Culture Collection (ATCC, Manassas, VA, USA). Cells were maintained at 37 °C in RPMI 1640 medium supplemented with 10% FBS, streptomycin (100 U/mL), and penicillin (100 U/mL). Cells were cultured in the presence of the SMR peptide for 24 h or 48 h, after which they were treated with various peptides—including SMRwt and SMRmut—to assess the effects of these treatments on the cells.

### 4.2. Antibodies

The following antibodies were used: (i) rabbit polyclonal and murine monoclonal anti-GFP antibodies (Cat #ab290, Abcam, Inc., Cambridge, MA, USA); (ii) rabbit polyclonal anti-Grp75 antibody (ab53098, Abcam, Inc. Cambridge, MA, USA); (iii) mouse monoclonal anti-α-tubulin antibody (Cat #T8203, Sigma-Aldrich, Inc., St. Louis, MO, USA); (iv) anti-Vimentin antibody (Cat #ab20346, Abcom, Inc., Cambridge, MA, USA); (v) goat anti-rabbit IgG (H + L) labeled with horseradish peroxidase antibody (Cat #31466, Thermo Fisher Scientific, Rockford, IL, USA); and (vi) HRP-coupled goat anti-mouse IgG (H + L) antibody (Thermo Fisher Scientific, Rockford, IL, USA).

### 4.3. SMR Peptides

The SMRwt peptide is a 5-mer peptide with a sequence of ^66^Val-Gly-Phe-Pro-Val^70^. The SMRmut peptide is a 5-mer peptide with a sequence of ^66^Ala-Gly-Phe-Pro-Val^70^. The difference between the two peptides is that the SMRmut peptide ^66^Val changes to an Ala residue in the same position. The SMRwt and SMRmut peptides were custom-made by INNOPEP Inc. (San Diego, CA, USA).

### 4.4. Proteins

Recombinant Human Vimentin Protein, CF (catalog # 2105-VI-100), Recombinant Human GRP75/HSPA9B/Mortalin His Protein (catalog # NBC1-18380), and Recombinant Human HSPA8/HSC71/Hsc70 His Protein (Catalog # NBPI-30278) were purchased from Novus Biologicals, LLC/R&D Systems, Centennial, CO, USA. These proteins were used for SPR.

### 4.5. Surface Plasmon Resonance (SPR)

SPR experiments were conducted using a Biacore T200 instrument (Cytiva, Marlborough, MA, USA). CM5 sensor chips (Cytiva) were used to immobilize proteins and run subsequent binding assays at 25 °C. To investigate the direct binding of Nef and SMR peptides to Vimentin and Mortalin and SMR competition studies, both proteins were immobilized as ligands on the active flow cells (FCs) using standard amine coupling chemistry. One reference FC was prepared using the same surface chemistry, and no proteins were immobilized onto this reference FC. Vimentin was immobilized to a level of ~2500 RU to 7200 RU in the presence of 4 mM of HCl and Mortalin to a level of ~11,900 RU to 13,200 RU in the presence of 10 mM of sodium acetate buffer at pH 4.5. Analytes were then injected (Nef, SMR peptides, Mortalin peptides, and mixtures of SMR peptides with SMR peptides). One 20 s pulse of 1:500 H_3_PO_4_ (H_3_PO_4_:ddH_2_O) was injected for surface regeneration. To investigate the direct binding of Mortalin peptides to Nef, Nef was immobilized as a ligand to ~450 RU using thiol coupling chemistry. A reference FC was created using a method similar to the one above. Mortalin peptides were then injected as analytes. One 20 s pulse of 1 M NaCl was injected for surface regeneration. HBS-P (10 mM Hepes, pH 7.4; 150 mM NaCl; 0.05% surfactant P20) was used as the immobilization running buffer during the immobilization of all ligands. HBS-P was also used to dilute analytes and as a running buffer during all analyte–ligand binding assays. SPR data used for analysis was both blank (signals due to HBS-P only) and reference-subtracted (signals corresponding to reference FCs). Sensorgrams recorded for kinetics analyses were fitted to a 1:1 binding model using the Biacore T200 evaluation software version 3.2.1. GraphPad Prism version 10.4.0 was used to plot all graphs.

### 4.6. Cell Transfection and Transfections with Peptides

Co-transfections of cells with peptides were carried out using Lipofectamine Reagent (Fisher Scientific), following the manufacturer’s protocol. One day prior to transfection, cells were trypsinized and plated at a density of 6.25 × 10^5^ cells per well in 2 mL of complete growth medium. Cell density should be 80% confluent on the day of transfection. On the day of transfection, the growth medium was removed from the cells and replaced with 2 mL of complete growth medium. For each well of cells, media and DNA-mix solution were prepared. Using a fresh 1.5 mL tube, 2.5 µg of Nef-GFP plasmid DNA was added, and either 200 ng of SMRwt-FLAG peptide or SMRmut-FLAG peptide and 4.5 µL of Lipofectamine Reagent were added to the 500 µL RPMI 1640 media, gently mixed, and incubated for 30 min at room temperature to form DNA–peptide Lipofectamine Reagent complexes. After 30 min of incubation, the media was removed from the cell culture. In total, 500 µL of the DNA–peptide Lipofectamine Reagent complexes was added directly to each well containing the cells and mixed gently. Complexes did not have to be removed following transfection. Then, 1.5 mL of complete growth medium (10% FBS) was added. Cells were incubated at 37 °C in a CO_2_ incubator for 18–24 h post transfection before transgene expression was assayed. The cell transfection efficiency was calculated by counting the green, fluorescent cells using a fluorescence microscope.

### 4.7. Co-Immunoprecipitation (Co-IP)

MDA-MB-231, MCF-7, and BT474 BC cells were grown in RPMI 1640 medium containing 10% FBS at 37 °C in a T-75 tissue culture flask and collected via centrifugation at 1000× *g* for 20 min at 4 °C. The cell pellet was resuspended in 1 mL of 1X PBS and transferred to a 1.5 mL Eppendorf tube. Following microcentrifugation at 1000× *g* for 1 min, the pellet was resuspended in 1 mL of α-FLAG Lysis buffer through gentle pipetting (50 mM Tris HCl, pH 7.4; 150 mM sodium chloride [NaCl]; 1 mM EDTA; and 1% Triton X-100, with 1 mM phenylmethyl sulfonyl fluoride [PMSF] and 10 µL/mL Protease Inhibitor Cocktail with mammalian cell and tissue extracts [PIC; Sigma-Aldrich] added immediately before use). The suspension was differentially microcentrifuged at 2000× *g* for 5 min and at 13,000× *g* for 10 min to pellet the cell debris and DNA, respectively, and the supernatant (hereafter referred to as the Lysate) was collected. In total, 1 milligram of Lysate protein and 1 milligram of the SMRwt or SMRmut peptide were added to 20 µL of Anti-FLAG^®^ M2 Affinity Gel (resin; Sigma). The mixture was brought to a final volume of 1 mL in Co-IP buffer (α-FLAG Lysis buffer minus PMSF and PIC) and incubated at 4 °C overnight with gentle rocking. The Lysate was also incubated with the affinity resin without either peptide as a negative control. The resin was pelleted via microcentrifugation at 5900× *g* for 30 s and allowed to settle for 2 min before supernatant aspiration. The resin was washed four times with 500 µL of wash buffer (50 mM Tris HCl, pH 7.4, and 150 mM NaCl) before eluting the bound cellular proteins with 50 µL of wash buffer containing 15 µg of 3X FLAG peptide (Sigma). The eluates were concentrated via acetone precipitation, boiled in Laemmli sample buffer (Bio-Rad laboratories Inc., Hercules, CA, USA), and separated using SDS-PAGE on a 4–20% Tris-HCl Criterion precast gel (Bio-Rad laboratories Inc.).

### 4.8. Western Immunoblot Analysis

Cell proteins were analyzed using Western blot analysis, as described previously [20,22]. The protein samples were separated using SDS-PAGE on 4–20% Tris-HCl Criterion precast gels (Bio-Rad) and transferred to the nitrocellulose membrane electrophoretically. The membrane was washed in Tris-Buffered Saline (TBS; Bio-Rad) for 5 min, blocked with 5% nonfat milk in TTBS (TBS with 0.1% Tween 20) for 1 h via shaking at room temperature, and processed for immunoblotting using a specific primary antibody with shaking at 4 °C overnight, followed by a secondary HRP-conjugated IgG (H + L) antibody. Protein bands were detected using Western blotting Luminol Reagent (Santa Cruz Biotechnology, Inc., Santa Cruz, CA, USA). Densitometry analysis was performed using ImageJ (National Institutes of Health, Bethesda, MD, USA).

### 4.9. Exosome Isolation and Purification

As previously reported [22], treated and untreated BC cell culture supernatants underwent sequential centrifugations: 400× *g* for 10 min, 10,000× *g* for 30 min, and 200,000× *g* for 2 h. The pelleted exosomes were washed, resuspended with PBS, and stored at 4 °C until used for Nanosight analysis or resuspended in 1X Lysis buffer (Thermo Fisher Scientific, Waltham, MA, USA) for Western blot analysis.

### 4.10. Statistical Analysis

Data are presented as the means ± standard deviations (SDs) of three independent experiments. One-way analysis of variance (ANOVA) was used to compare the groups, followed by Tukey’s multiple comparison tests using the statistics module of GraphPad Prism. A *p*-value of <0.05 was considered statistically significant.

## 5. Conclusions

Understanding peptide–protein interactions is crucial for elucidating cancer metastasis and HIV-1 pathogenesis mechanisms. These findings have significant implications for the development of targeted therapies to disrupt these interactions and inhibit disease progression. Future research should focus on further characterizing these interactions and exploring their therapeutic potential in greater detail.

## Figures and Tables

**Table 1:** Summary of K_D_ values (affinity, K_D_).

Analyte	Ligand	
	Vimentin K_D_	Mortalin K_D_
SMRwt	6.63 +/− 0.74 µM	20.73 +/− 2.33 µM
SMRmut	5.03 +/− 2.12 µM	NB
Nef	0.75 +/− 1.1 nM	3.16 +/− 0.03 nM

NB: No meaningful binding was measured within the tested dose range.

## Data Availability

Information on the chemicals used in this manuscript may be requested from the corresponding authors.

## Supplementary Materials

The following supporting information can be downloaded at: https://www.mdpi.com/article/10.3390/ijms26188848/s1.

## Abbreviations

BC	Breast Cancer
EV	Extracellular Vesicle
SPR	Surface Plasmon Resonance
SMR	Secretion Modification Region
EMT	Epithelial–Mesenchymal Transition

## References

[1] Marino N., Woditschka S., Reed L.T., Nakayama J., Mayer M., Wetzel M., Steeg P.S. (2013). Breast cancer metastasis: Issues for the personalization of its prevention and treatment. Am. J. Pathol..

[2] Liu T., Hooda J., Atkinson J.M., Whiteside T.L., Oesterreich S., Lee A.V. (2021). Exosomes in Breast Cancer—Mechanisms of Action and Clinical Potential. Mol. Cancer Res..

[3] Wang X., Sun C., Huang X., Li J., Fu Z., Li W., Yin Y. (2021). The Advancing Roles of Exosomes in Breast Cancer. Front. Cell Dev. Biol..

[4] Lakshmi S., Hughes T.A., Priya S. (2021). Exosomes and exosomal RNAs in breast cancer: A status update. Eur. J. Cancer..

[5] Yuan X., Qian N., Ling S., Li Y., Sun W., Li J., Du R., Zhong G., Liu C., Yu G. (2021). Breast cancer exosomes contribute to pre-metastatic niche formation and promote bone metastasis of tumor cells. Theranostics.

[6] Piombino C., Mastrolia I., Omarini C., Candini O., Dominici M., Piacentini F., Toss A. (2021). The Role of Exosomes in Breast Cancer Diagnosis. Biomedicines.

[7] Wang Y., Ma Q., Wang T., Xing J., Li Q., Wang D., Wang G. (2024). The involvement and application potential of exosomes in breast cancer immunotherapy. Front. Immunol..

[8] Kok V.C., Yu C.C. (2020). Cancer-Derived Exosomes: Their Role in Cancer Biology and Biomarker Development. Int. J. Nanomed..

[9] Wang H.X., Gires O. (2019). Tumor-derived extracellular vesicles in breast cancer: From bench to bedside. Cancer Lett..

[10] Das A., Mohan V., Krishnaswamy V.R., Solomonov I., Sagi I. (2019). Exosomes as a storehouse of tissue remodeling proteases and mediators of cancer progression. Cancer Metastasis Rev..

[11] Vlassov A.V., Magdaleno S., Setterquist R., Conrad R. (2012). Exosomes: Current knowledge of their composition, biological functions, and diagnostic and therapeutic potentials. Biochim. Biophys. Acta..

[12] Deng B., Huang W., Tan Q.Y., Fan X.Q., Jiang Y.G., Liu L., Zhong Y.Y., Liang Y.G., Wang R.W. (2011). Breast cancer anti-estrogen resistance protein 1 (BCAR1/p130cas) in pulmonary disease tissue and serum. Mol. Diagn. Ther..

[13] Deslouches B., Di Y.P. (2017). Antimicrobial peptides with selective antitumor mechanisms: Prospect for anticancer applications. Oncotarget.

[14] E-Kobon T., Thongararm P., Roytrakul S., Meesuk L., Chumnanpuen P. (2016). Prediction of anticancer peptides against MCF-7 breast cancer cells from the peptidomes of Achatina fulica mucus fractions. Comput. Struct. Biotechnol. J..

[15] Marqus S., Pirogova E., Piva T.J. (2017). Evaluation of the use of therapeutic peptides for cancer treatment. J. Biomed. Sci..

[16] Chu H.L., Yip B.S., Chen K.H., Yu H.Y., Chih Y.H., Cheng H.T., Chou Y.T., Cheng J.W. (2015). Novel antimicrobial peptides with high anticancer activity and selectivity. PLoS ONE.

[17] Chih Y.H., Lin Y.S., Yip B.S., Wei H.J., Chu H.L., Yu H.Y., Cheng H.T., Chou Y.T., Cheng J.W. (2015). Ultrashort Antimicrobial Peptides with Antiendotoxin Properties. Antimicrob. Agents Chemother..

[18] Lumongga F., Dharmajaya R., Siregar K., Delyuzar D., Handjari D., Jusuf N., Munir D., Asrul A. (2022). Correlation Between Intensity of Vimentin Immuno-expression in Young Women with Triple Negative Breast Cancer and Its Cliniocopathological Parameters. Med. Arch..

[19] Grasset E.M., Dunworth M., Sharma G., Loth M., Tandurella J., Cimino-Mathews A., Gentz M., Bracht S., Haynes M., Fertig E.J. (2022). Triple-negative breast cancer metastasis involves complex epithelial-mesenchymal transition dynamics and requires vimentin. Sci. Transl. Med..

[20] Zhang H., Wang Y., Liu C., Li W., Zhou F., Wang X., Zheng J. (2022). The Apolipoprotein C1 is involved in breast cancer progression via EMT and MAPK/JNK pathway. Pathol. Res. Pract..

[21] Thalla D.G., Jung P., Bischoff M., Lautenschlager F. (2021). Role of Extracellular Vimentin in Cancer-Cell Functionality and Its Influence on Cell Monolayer Permeability Changes Induced by SARS-CoV-2 Receptor Binding Domain. Int. J. Mol. Sci..

[22] Chen Z., Fang Z., Ma J. (2021). Regulatory mechanisms and clinical significance of vimentin in breast cancer. Biomed. Pharmacother..

[23] Fan J.Q., Li Y.J., Wei Z.J., Fan Y., Li X.D., Chen Z.M., Hou D.Y., Xiao W.Y., Ding M.R., Wang H. (2021). Binding-Induced Fibrillogenesis Peptides Recognize and Block Intracellular Vimentin Skeletonization against Breast Cancer. Nano Lett..

[24] Korsching E., Packeisen J., Liedtke C., Hungermann D., Wulfing P., van Diest P.J., Brandt B., Boecker W., Buerger H. (2005). The origin of vimentin expression in invasive breast cancer: Epithelial-mesenchymal transition, myoepithelial histogenesis or histogenesis from progenitor cells with bilinear differentiation potential?. J. Pathol..

[25] Zhao Q., He Y. (2020). Challenges of Convalescent Plasma Therapy on COVID-19. J. Clin. Virol..

[26] Li H., Zhu W., Zhang L., Lei H., Wu X., Guo L., Chen X., Wang Y., Tang H. (2015). The metabolic responses to hepatitis B virus infection shed new light on pathogenesis and targets for treatment. Sci. Rep..

[27] Arrindell J., Desnues B. (2023). Vimentin: From a cytoskeletal protein to a critical modulator of immune response and a target for infection. Front. Immunol..

[28] Berr A.L., Wiese K., Dos Santos G., Koch C.M., Anekalla K.R., Kidd M., Davis J.M., Cheng Y., Hu Y.S., Ridge K.M. (2023). Vimentin is required for tumor progression and metastasis in a mouse model of non-small cell lung cancer. Oncogene.

[29] Satelli A., Li S. (2011). Vimentin in cancer and its potential as a molecular target for cancer therapy. Cell Mol. Life Sci..

[30] Park J.I. (2023). Editorial: The role of mortalin in biology and disease. Front. Cell Dev. Biol..

[31] Londono C., Osorio C., Gama V., Alzate O. (2012). Mortalin, apoptosis, and neurodegeneration. Biomolecules.

[32] Esfahanian N., Knoblich C.D., Bowman G.A., Rezvani K. (2023). Mortalin: Protein partners, biological impacts, pathological roles, and therapeutic opportunities. Front. Cell Dev. Biol..

[33] Yoon A.R., Wadhwa R., Kaul S.C., Yun C.O. (2022). Why is Mortalin a Potential Therapeutic Target for Cancer?. Front. Cell. Dev. Biol..

[34] Wei B., Cao J., Tian J.H., Yu C.Y., Huang Q., Yu J.J., Ma R., Wang J., Xu F., Wang L.B. (2021). Mortalin maintains breast cancer stem cells stemness via activation of Wnt/GSK3beta/beta-catenin signaling pathway. Am. J. Cancer Res..

[35] Kim S.Y., Byrn R., Groopman J., Baltimore D. (1989). Temporal aspects of DNA and RNA synthesis during human immunodeficiency virus infection: Evidence for differential gene expression. J. Virol..

[36] Campbell T.D., Khan M., Huang M.B., Bond V.C., Powell M.D. (2008). HIV-1 Nef protein is secreted into vesicles that can fuse with target cells and virions. Ethn Dis..

[37] Ali S.A., Huang M.B., Campbell P.E., Roth W.W., Campbell T., Khan M., Newman G., Villinger F., Powell M.D., Bond V.C. (2010). Genetic characterization of HIV type 1 Nef-induced vesicle secretion. AIDS Res. Hum. Retroviruses.

[38] Jin H., Ji M., Chen L., Liu Q., Che S., Xu M., Lin Z. (2016). The clinicopathological significance of Mortalin overexpression in invasive ductal carcinoma of breast. J. Exp. Clin. Cancer Res..

[39] Masoud V., Pages G. (2017). Targeted therapies in breast cancer: New challenges to fight against resistance. World J. Clin. Oncol..

[40] Elwakeel A. (2022). Abrogating the Interaction Between p53 and Mortalin (Grp75/HSPA9/mtHsp70) for Cancer Therapy: The Story so far. Front. Cell Dev. Biol..

[41] Rai R., Kennedy A.L., Isingizwe Z.R., Javadian P., Benbrook D.M. (2021). Similarities and Differences of Hsp70, hsc70, Grp78 and Mortalin as Cancer Biomarkers and Drug Targets. Cells.

[42] Wu P.K., Hong S.K., Starenki D., Oshima K., Shao H., Gestwicki J.E., Tsai S., Park J.I. (2020). Mortalin/HSPA9 targeting selectively induces KRAS tumor cell death by perturbing mitochondrial membrane permeability. Oncogene.

[43] Wadhwa R., Colgin L., Yaguchi T., Taira K., Reddel R.R., Kaul S.C. (2002). Rhodacyanine dye MKT-077 inhibits in vitro telomerase assay but has no detectable effects on telomerase activity in vivo. Cancer Res..

[44] Wadhwa R., Sugihara T., Yoshida A., Nomura H., Reddel R.R., Simpson R., Maruta H., Kaul S.C. (2000). Selective toxicity of MKT-077 to cancer cells is mediated by its binding to the hsp70 family protein mot-2 and reactivation of p53 function. Cancer Res..

[45] Alfadhalah T., Elamir H. (2019). Exploring leadership styles in government hospitals in Kuwait. Leadersh Health Serv..

[46] Barlin M., Erdmann-Gilmore P., Mudd J.L., Zhang Q., Seymour R.W., Guo Z., Miessner J.R., Goedegebuure S.P., Bi Y., Osorio O.A. (2023). Proteins in Tumor-Derived Plasma Extracellular Vesicles Indicate Tumor Origin. Mol. Cell Proteom..

[47] Huang M.B., Gonzalez R.R., Lillard J., Bond V.C. (2017). Secretion modification region-derived peptide blocks exosome release and mediates cell cycle arrest in breast cancer cells. Oncotarget.

[48] Huang M.B., Wu J.Y., Lillard J., Bond V.C. (2019). SMR peptide antagonizes mortalin promoted release of extracellular vesicles and affects mortalin protection from complement-dependent cytotoxicity in breast cancer cells and leukemia cells. Oncotarget.

[49] Huang M.B., Brena D., Wu J.Y., Roth W.W., Owusu S., Bond V.C. (2022). Novel secretion modification region (SMR) peptide exhibits anti-metastatic properties in human breast cancer cells. Sci. Rep..

[50] Miller-Kleinhenz J., Guo X., Qian W., Zhou H., Bozeman E.N., Zhu L., Ji X., Wang Y.A., Styblo T., O’Regan R. (2018). Dual-targeting Wnt and uPA receptors using peptide conjugated ultra-small nanoparticle drug carriers inhibited cancer stem-cell phenotype in chemo-resistant breast cancer. Biomaterials.

[51] Peess C., von Proff L., Goller S., Andersson K., Gerg M., Malmqvist M., Bossenmaier B., Schraml M. (2015). Deciphering the stepwise binding mode of HRG1beta to HER3 by surface plasmon resonance and interaction map. PLoS ONE.

[52] Douzi B. (2017). Protein-Protein Interactions: Surface Plasmon Resonance. Methods Mol. Biol..

[53] Kumar M., Gouw M., Michael S., Samano-Sanchez H., Pancsa R., Glavina J., Diakogianni A., Valverde J.A., Bukirova D., Calyseva J. (2020). ELM-the eukaryotic linear motif resource in 2020. Nucleic Acids Res..

[54] Dinkel H., Van Roey K., Michael S., Kumar M., Uyar B., Altenberg B., Milchevskaya V., Schneider M., Kuhn H., Behrendt A. (2016). ELM 2016--data update and new functionality of the eukaryotic linear motif resource. Nucleic Acids Res..

[55] Kumar M., Michael S., Alvarado-Valverde J., Zeke A., Lazar T., Glavina J., Nagy-Kanta E., Donagh J.M., Kalman Z.E., Pascarelli S. (2024). ELM-the Eukaryotic Linear Motif resource-2024 update. Nucleic Acids Res..

[56] Drescher D.G., Selvakumar D., Drescher M.J. (2018). Analysis of Protein Interactions by Surface Plasmon Resonance. Adv. Protein Chem. Struct. Biol..

[57] Myszka D.G. (1997). Kinetic analysis of macromolecular interactions using surface plasmon resonance biosensors. Curr. Opin. Biotechnol..

[58] Hahnefeld C., Drewianka S., Herberg F.W. (2004). Determination of kinetic data using surface plasmon resonance biosensors. Methods Mol. Med..

[59] Kabakov A.E., Gabai V.L. (2021). HSP70s in Breast Cancer: Promoters of Tumorigenesis and Potential Targets/Tools for Therapy. Cells.

[60] Na Y., Kaul S.C., Ryu J., Lee J.S., Ahn H.M., Kaul Z., Kalra R.S., Li L., Widodo N., Yun C.O. (2016). Stress chaperone mortalin contributes to epithelial-mesenchymal transition and cancer metastasis. Cancer Res..

