# Supplementary material for: Investigating SMR Peptide Interactions with Breast Cancer-Associated Proteins
Source: Int J Mol Sci. 2025 Sep 11;26(18):8848. doi: 10.3390/ijms26188848 (PMC12469269; doi:10.3390/ijms26188848)
Supplement: Supplementary file 1 [file ijms-26-08848-s001.zip › ijms-3689756-supplementary.pdf]

Results

2.11. Development and Testing of Mortalin-Derived Scanning Peptides for Effects on EV Secretion

To identify peptide sequences within Mortalin that modulate extracellular vesicle (EV) secretion, a panel of scanning peptides was synthesized and tested in Jurkat T cells. The peptides, listed in Table 1, were custom synthesized with an average purity of 75% and used in transfection assays to evaluate their impact on EV release.

Jurkat T cells (3×10^5) were co-transfected with 50 ng of individual Mortalin peptides or 250 ng of wild-type Nef-GFP plasmid using Chariot™ transfection reagent. Transfection efficiency was confirmed by fluorescence microscopy after 48 hours. EV secretion was assessed by measuring GFP fluorescence in the culture media, and statistical analysis was performed using unpaired two-factor t-tests ($p < 0.05$). The peptide sequences and experimental conditions are summarized in Table 1.

Table S1. Panel of Mortalin scanning peptides developed and tested for their effect EV secretion

No	Sequence	No	Sequence	No	Sequence
1	MISASRAAAARLVGAAASRG	24	GLDKSEDKVIAVYDLGGGTF	47	NTTIPTKKSQVFSTAADGQT
2	RLVGAAASRGPTAARHQDSW	25	AVYDLGGGTFDISILEIQKG	48	VFSTAADGQTQVEIKVCQGE
3	PTAARHQDSWNGLSHEAFRL	26	DISILEIQKGVFEVKSTNGD	49	QVEIKVCQGEREMAGDNKLL
4	NGLSHEAFRLVSRRDYASEA	27	VFEVKSTNGDTFLGGEDFDQ	50	REMAGDNKLLGOFTLIGIPP
5	VSRRDYASEAIKGAVVGIDL	28	TFLGGEDFDQALLRHIVKEF	51	GQFTLIGIPPAPRGVPQIEV
6	IKGAVVGIDLGTTNSCVAVM	29	ALLRHIVKEFKRETGVDLTK	52	APRGVPQIEVTFDIDANGIV
7	GTINSCVAVMEGKRAKVLEN	30	KRETGVDLTKDNMALQRVRE	53	TFDIDANGIVHVSAKDKGTG
8	EGKRAKVLENAEGARTTPSV	31	DNMALQRVREAAEKAKCELS	54	HVSAKDKGTGREQIVIQSS
9	AEGARTTPSVVAFTADGERL	32	AAEKAKCELSSSVQTDINLP	55	REQQIVIQSSGGLSKDDIEN
10	VAFTADGERLVGMPAKRQAV	33	SSVQTDINLPYLTMDSSGPK	56	GGLSKDDIENMVKNAEKYAE
11	VGMPAKRQAVTNPNNTFYAT	34	YLTMDSSGPKHLNMKLTRAQ	57	MVKNAEKYAEEDRRKKERVE
12	TFPNNTFYATKRLIGRRYDD	35	HLNMKLTRAQFEGIVTIDLIR	58	EDRRKKERVEAVNMAEGIIH
13	KRLIGRRYDDPEVQKDIKNV	36	FEGIVTDLIRRTIAPCQKAM	59	AVNMAEGIIHDTETKMEEFK
14	PEVQKDIKNVPFKIVRASNG	37	RTIAPCQKAMQDAEVSKSDI	60	DTETKMEEFKDQLPADECNK
15	PFKIVRASNGDAWVEAHGKL	38	QDAEVSKSDIGEVILVGGMT	61	DQLPADECNKLKEEISKMRE
16	DAWVEAHGKLYSPSQIGAFV	39	GEVILVGGMTRMPKVQOTVQ	62	LKEEISKMRELLARKDSETG
17	YSPSQIGAFVLMKMKETAEN	40	RMPKVQOTVQDLFGRAPSKA	63	LLARKDSETGENIRQAASSL
18	LMKMKKETAENYLGRTAKNAV	41	DLFGRAPSKAVNPDEAVAIG	64	ENIRQAASSLQOASLKLFEM
19	YLGRTAKNAVITVPAYFNDS	42	VNPDEAVAIGAAIQGGVLAG	65	QOASLKLFEMAYKKMASERE
20	ITVPAYFNDSQRQATKDAGQ	43	AAIQGGVLAGDVTDVLLLDV	66	AYKKMASEREGSGSSGTGEQ
21	QRQATKDAGQISGLNVLRVI	44	DVTDVLLLDVTPLSLGIETL	67	GSGSSGTGEQKEDQKEEKQ
22	ISGLNVLRVINEPTAAALAY	45	TPLSLGIETLGGVFTKLINR		
23	NEPTAAALAYGLDKSEDKVI	46	GGVFTKLINRNTTIPTKKSQ		

Underline denotes the nucleotide-binding domain (NBD).

Table S2. Mortalin Scanning Peptides Used in Transfection Assays

Peptide ID	Sequence (N→C)	Domain/Region
#56	KFERQKLLSEKA	Substrate-binding domain
#57	LLSEKAKLQRAK	Substrate-binding domain
#58	KLQRAKELQKAK	Substrate-binding domain
#59	ELQKAKQAKKAK	Substrate-binding domain
#60	QAKKAKKAKKAK	Substrate-binding domain
#61	KKAKKAKKAKKA	Substrate-binding domain
#62	KKAKKAKKAKKE	Substrate-binding domain
#63	KAKKAKKEKAKK	Substrate-binding domain
#64	AKKEKAKKEKAK	Substrate-binding domain

#65	KEKAKKEKAKKE	Substrate-binding domain
#66	KAKKEKAKKEKA	Substrate-binding domain
#67	AKKEKAKKEKAK	Substrate-binding domain

All peptides were synthesized with an average purity of 75% and used at 50 ng per transfection in Jurkat T cells. Transfection efficiency was assessed by fluorescence microscopy, and EV secretion was quantified using GFP fluorescence. Statistical analysis was performed using unpaired two-factor t-tests ($p < 0.05$).

2.12. Identification of a Vimentin-Derived Peptide with Homology to the Mortalin SMR Binding Sequence

To identify a Vimentin-derived peptide with potential functional similarity to the Mortalin SMR binding sequence, a computational search was performed using the Mortalin SMR motif (LKEEISKMRE) as a query. The search revealed a homologous sequence within the human Vimentin protein, highlighted in Table 3, which closely resembles the Mortalin template.

Based on this alignment, the corresponding Vimentin peptide was custom synthesized by InnoPep (San Diego, CA, USA) for use in subsequent transfection and EV secretion assays.

VimWt-CPPtat: H-LYEEEMREGRKKRRQRRRPPQ-OH

Vim-Wt **CPPtat**

Vimentin Sequence (466 AA)

Vimentin: >sp Po8670 VIME_HUMAN Vimentin OS=Homo sapiens GN=VIM

MSTRSVSSSSYRRMFGGPGTASRPSSSRSYVTTSTRTYSLGSALRPSTSRSLYASSPGGVYA
TRSSAVRLRSSVPGVRLLQDSVDFSLADAINTEFKNTRTNEKVELQELNDRFANYIDKVRFL
EQQNKILLAELEQLKGQGKSRLGD**LYEEEMRE**LRRQVDQLTNDKARVEVERDNLAEDIM
RLREKLQEEMLQREEAENTLQSFRQDVDNASLARLDLERKVESLQEEIAFLKKLHEEEIQEL
QAQIQEQHVQIDVDVSKPDLTAALRDVRQQYESVAAKNLQEAEEWYKSKFADLSEAANR
NNDALRQAKQESTEYRRQVQSLTCEVDALKGTNESLERQMREMEENFAVEAANYQDTIG
RLQDEIQNMKEEMARHLREYQDLLNVKMALDIEIATYRKLLEGEESRISLPLPNFSSLNLRE
TNLDSLPLVDTHSKRTLLIKTVETRDGQVINETSQHHDDLE

Table S3. Identification of a Vimentin-Derived Peptide Homologous to the Mortalin SMR Binding Sequence

A computational sequence alignment was performed to identify regions within the human Vimentin protein that share homology with the Mortalin SMR binding motif (LKEEISKMRE). The search revealed a closely related sequence within Vimentin, which is highlighted in the table. This homologous peptide was custom synthesized by InnoPep (San Diego, CA, USA) for use in downstream functional assays.
